# Respiratory virus-associated infections in HIV-infected adults admitted to the intensive care unit for acute respiratory failure: a 6-year bicenter retrospective study (*HIV*-*VIR study*)

**DOI:** 10.1186/s13613-020-00738-9

**Published:** 2020-09-14

**Authors:** Alexandre Elabbadi, Jérémie Pichon, Benoit Visseaux, Aurélie Schnuriger, Lila Bouadma, Quentin Philippot, Juliette Patrier, Vincent Labbé, Stéphane Ruckly, Muriel Fartoukh, Jean-François Timsit, Guillaume Voiriot

**Affiliations:** 1 Assistance Publique – Hôpitaux de Paris, Service de médecine intensive réanimation, Hôpital Tenon, Sorbonne Université, Paris, France; 2 Assistance Publique – Hôpitaux de Paris, Service de virologie, Hôpital Bichat, Université de Paris, Paris, France; 3 UMR 1137-IAME Team 5-DeSCID: Decision SCiences in Infectious Diseases control and care, INSERM, Université de Paris, Paris, France; 4 INSERM, Saint-Antoine Research Center (CRSA), Assistance Publique – Hôpitaux de Paris, Département de virologie site Trousseau, Sorbonne Université, Paris, France; 5 Assistance Publique – Hôpitaux de Paris, Service de réanimation médicale et infectieuse, Hôpital Bichat, Université de Paris, Paris, France; 6 Groupe de Recherche Clinique GRC05 CARMAS, Institut Mondor de recherche biomédicale, INSERM, Université Paris Est, Créteil, France

**Keywords:** Acute respiratory failure, Human immunodeficiency virus, Polymerase chain reaction, Viral pneumonia, Viral pneumonia

## Abstract

**Introduction:**

Acute respiratory failure is the main reason for admission to the intensive care unit (ICU) in HIV-infected adults. There is little data about the epidemiology of respiratory viruses in this population.

**Methods:**

HIV-infected adults admitted to two intensive care units over a 6-year period for an acute respiratory failure and explored for respiratory viruses with multiplex polymerase chain reaction (mPCR) were retrospectively selected. Objectives were to describe the prevalence of respiratory viruses, coinfections with non-viral pathogens, and hospital outcome.

**Results:**

A total of 123 episodes were included. An HIV infection was newly diagnosed in 9% of cases and 72% of the population were on antiretroviral therapy. Real-time mPCR tests identified at least one respiratory virus in the respiratory tract of 33 (27%) patients, but with a non-viral copathogen in two-thirds of cases. Rhinovirus was predominant, documented in 15 patients, followed by Influenza and Respiratory Syncytial Viruses (both *n* = 6). The prevalence of respiratory virus-associated infection did not vary along with the level of the CD4 T-cell deficiency, except for Rhinovirus which was more prevalent in patients with a CD4 lymphocyte count below 200 cells/µL (*n* = 13 (20%) vs. *n* = 2 (4%), *p* < 0.01). In multivariate analysis, respiratory virus-associated infection was not associated with a worse prognosis.

**Conclusions:**

Viruses are frequently identified in the respiratory tract of HIV-infected patients with acute respiratory failure that requires ICU admission, but with a non-viral copathogen in two-thirds of cases. Rhinovirus is the predominant viral specie; its prevalence is highest in patients with a CD4 lymphocyte count below 200 cells/µL.

## Introduction

Acute respiratory failure (ARF) is the leading cause of admission to the intensive care unit (ICU) in HIV-infected patients [1–3]. Infectious causes are predominant, although the proportion of opportunistic infections has decreased significantly in the era of combination antiretroviral therapy (ART) [2, 4, 5]. In contrast, the burden of non-HIV-related pulmonary events, such as bacterial pneumonia, acute bronchitis and acute exacerbation of chronic obstructive pulmonary disease (COPD) has been shown increasing [2, 3, 6]. These important changes in the etiologic panel of ARF in HIV-infected patients question the role of respiratory viruses. Indeed, using nucleic acid amplification test such as multiplex polymerase chain reaction (mPCR), these pathogens have been shown highly prevalent (20–56%) in large cohorts of adult patients admitted to the ICU for all-cause ARF [7, 8], community-acquired pneumonia [9, 10], hospital-acquired pneumonia [11], acute exacerbation of COPD [12, 13], and asthma [14], compared to asymptomatic adults [15, 16]. High prevalence has also been described in specific immunocompromised populations, such as cancer and hematology patients [17, 18]. In contrast, little is known about the epidemiology of respiratory viruses in HIV-infected patients [19, 20], especially those admitted to the ICU, and the prevalence of respiratory viruses according to the CD4 T-cell deficiency. Moreover, coinfections with virus and opportunistic pathogens may occur. Overall, respiratory virus-associated infections may affect prognosis.

Therefore, we conducted a comprehensive observational study among adult HIV-infected ICU patients with ARF explored with respiratory mPCR. Our goals were to describe the prevalence of respiratory viruses, coinfections with non-viral pathogens, and hospital outcome.

## Methods

### Study design and patient selection

We conducted a retrospective bicenter observational study in two ICU of the Paris area (the 26-bed ICU of the Bichat University Hospital and the 20-bed ICU of the Tenon University Hospital). From April 2011 to April 2017, all consecutive HIV-infected patients admitted to ICU having undergone an mPCR in the respiratory tract within 72 h following their ICU admission were screened. Medical records were independently reviewed by two physicians (AE and GV). All patients with ARF at ICU admission were included. ARF was defined by the presence of at least two of the following criteria: cough, expectoration, dyspnea, rales, signs of respiratory distress (tachypnea exceeding 30/min, paradoxical abdominal breathing), chest pain, hypoxemia requiring oxygen therapy, noninvasive ventilation or intubation. In case of multiple admissions over the 6-year study period, only the first admission was analyzed.

### Data collection

At ICU admission and during ICU stay, data regarding demographics, comorbidity, HIV-related characteristics, clinical examinations, laboratory and radiological findings, microbiologic investigations, and therapeutic management were collected (for details, see Additional file 1). Mortality was defined as death from any cause within 28 days following the ICU admission.

### Microbiological evaluation

Respiratory mPCRs were performed either in nasopharyngeal (NP) swabs or in lower respiratory tract (LRT) specimen, usually bronchoalveolar lavage (BAL) fluid otherwise endotracheal aspirate. During the study period, different respiratory mPCR kits (Additional file 1: Table S1) were used (for more details about microbiological evaluation, see Additional file 1).

### Classification of patients according to the causative diagnosis of ARF

Medical charts were independently reviewed by two clinicians (AE and GV). They determined the causative diagnosis of ARF for each patient, using a 5-class classification. In case of an inter-reviewer discordance, a shared review of the medical charts was performed, and an agreement was found. The five mutually exclusive classes of causative diagnosis for ARF were: (i) *Pneumocystis jirovecii* pneumonia (PCP); (ii) other opportunistic lung infections; (iii) non-opportunistic acute lung infection; (iv) non-infectious lung disease, and (v) extra-pulmonary cause (for details, see Additional file 1).

### Endpoints

The primary endpoint was to determine the prevalence of respiratory viruses according to the CD4 lymphocyte count. A respiratory virus documented with mPCR was always considered as a pathogen of the respiratory tract, regardless of the type of specimen (NP or LRT). The CD4 lymphocyte count measured during the ICU stay was used to group patients, with a cut-off of 200 cells/µL (≤ 200 cells/µL for the Low-CD4 group; > 200 cells/µL for the High-CD4 group) [19, 21].

Secondary endpoints were to describe the epidemiology of respiratory viruses and coinfections with non-viral pathogens, to identify risk factors for respiratory virus-associated infection, and to study outcome. A composite criterion named “complicated course” included death from any cause within 28 days following the ICU admission or mechanical ventilation for more than 7 days.

### Data presentation and statistical analysis

Continuous data were expressed as median [first through third quartiles] and were compared using the pairwise Mann and Whitney test. Categorical data were expressed as number (percentage) and were evaluated using the chi-square test or Fisher exact test. *p* values less than 0.05 were considered significant. A univariate logistic regression with clinically relevant variables was used to identify variables associated with a respiratory virus-associated infection. A multivariate conditional logistic regression, including variables with *p* value less than 0.10 in the previous step, was used to identify variables independently associated with a respiratory virus-associated infection. Similar statistical analyses were performed to identify variables independently associated with death from any cause within 28 days following the ICU admission and mechanical ventilation for more than 7 days in survivors at Day-28. Quantitative variables that did not validate the log-linearity assumption were transformed into categorical variables according to their median value. Missing data were imputed to the median or the more frequent value. The accuracy of the final model was tested using area under the receiver operating characteristic curve analysis and the Hosmer–Lemeshow chi-square test. Analyses were performed using the SAS software package (SAS Institute, Cary, NC, USA).

### Ethical considerations

This study was approved by the institutional review board of the French Society of Respiratory Diseases (*Reference CEPRO 2018*-*017*) according to the French regulations. The board waived the need for signing consent for patients included in the study.

## Results

### Population

During the 6-year study period, 135 HIV-infected adult patients were admitted at least once to ICU and underwent a respiratory mPCR in the first 72 h of the ICU stay. Among them, 12 did not fulfill criteria of ARF. The final study group consisted of 123 patients. Their main characteristics, stratified by the CD4 lymphocyte count at ICU admission, are presented in Table 1. Of these 123 patients, 2 were admitted twice during the study period and one was admitted thrice. Eleven patients (9%) were newly diagnosed as having HIV infection on ICU admission; the remaining 112 had been previously diagnosed, and 88 were on ART but with poor adherence to the treatment in 21 patients, as mentioned by the infectiologist in the medical charts. Latest available median CD4 lymphocyte count and HIV viral load were 351 cells/µL [140–600] and 0 log copies/mL [0–3.4], respectively. At least one additional factor of immunosuppression was identified in 10 (8%) patients.

**Table 1:** Baseline characteristics, behavior during ICU stay, and outcome of 123 HIV-infected patients admitted to the ICU for acute respiratory failure, according to the CD4 lymphocyte count on the ICU admission

Patients	All patients (*n* = 123)	CD4 ≤ 200 (*n* = 66)	CD4 > 200 (*n* = 57)	*p* value^a^
Age (year)	51 [43–59]	46 [39–56]	55 [47–59]	< 0.01
Sex male	82 (66.7)	40 (60.6)	42 (73.7)	0.12
Smoking	49 (41.2)	24 (38.7)	25 (43.9)	0.57
WHO performance status > 0	61 (50.8)	33 (50.7)	28 (50.9)	0.99
COPD GOLD III–IV	16 (13)	4 (6.1)	12 (21.1)	0.01
Arterial hypertension	32 (26)	13 (19.7)	19 (33.3)	0.09
Coronary heart disease	19 (15.4)	10 (15.2)	9 (15.8)	0.92
Baseline HIV-related characteristics				
Newly diagnosed HIV infection	11 (8.9)	10 (15.2)	1 (1.8)	0.92
HIV viral load (log)^b^	0 [0–3.4]	4.7 [1.2–5.4]	0 [0–1.4]	< 0.01
CD4 lymphocyte count (cells/µL)^c^	351 [140–600]	72 [30–200]	517 [406–715]	< 0.01
ART	88 (72.1)	33 (50.8)	55 (96.5)	< 0.01
Steroid therapy^d^	4 (3.3)	2 (3)	2 (3.5)	0.85
Other immunosuppressive treatments	2 (1.6)	1 (1.5)	1 (1.8)	0.92
Splenectomy	1 (0.8)	0	1 (1.8)	0.28
Cancer or hematologic malignancy	6 (4.9)	1 (1.5)	5 (8.8)	0.06
Chemotherapy	2 (1.6)	1 (1.5)	1 (1.8)	0.92
Organ/bone marrow transplantation	(0.8)	1 (1.5)	0	0.35
Transfer from another ward^e^	62 (50.4)	30 (45.5)	32 (56.2)	0.24
SOFA score	4 [2–7]	4 [2–8]	3 [2–6]	0.60
SAPS II score	44 [34–57]	44 [37–57]	41 [31–55]	0.20
Biology on ICU admission				
HIV viral load (log)^f^	2.5 [0–5.3]	5 [2.9–5.6]	0 [0–2]	< 0.01
CD4 lymphocyte count (cells/µL)	170 [20–430]	29 [10–102]	461[345–533]	< 0.01
Neutrophil count (G/L)	6.7 [3.9–9]	5.5 [2.1–7.6]	7.6 [5.3–12.1]	< 0.01
Procalcitonin (µg/L)^g^	0.6 [0.2–5.9]	0.6 [0.2–3]	0.5 [0.1–8.3]	0.89
Lactate dehydrogenase (U/L)	403 [276–637]	471 [325–675]	327 [233–575]	0.02
Organ supports during ICU stay				
High-flow nasal cannula oxygen	36 (29.2)	24 (36.3)	12 (21)	0.06
Noninvasive ventilation	30 (24.8)	11 (17.1)	19 (33.3)	0.04
Mechanical ventilation	43 (35.2)	24 (36.9)	19 (33.3)	0.68
Vasopressor	36 (29.3)	22 (33.3)	14 (24.6)	0.29
Renal replacement therapy	23 (18.7)	14 [21.2)	9 (15.8)	0.44
Outcome				
ICU length of stay (day)	7 [4–12]	7 [3.3–16.8]	6 [3–11]	0.21
Day-28 mortality^h^	15 (12.2)	8 (12.1)	7 (12.3)	0.98
Complicated course^i^	30 (24.4)	17 (25.8)	13 (22.8)	0.70

Data are presented as median [first through third quartiles] or number (%). CD4 refers to CD4 lymphocyte count (cells/µL)

*HIV* Human immunodeficiency virus, *ICU* Intensive care unit, *SAPS II* Simplified Acute Physiologic Score II, *SOFA* Sepsis-related Organ Failure Assessment, *WHO* World Health Organization

^a^ P values refer to differences between Low-CD4 (≤ 200 cells/µL) and High-CD4 (> 200 cells/µL) groups in univariate logistic regression

^b^Data were available for 76 patients

^c^Data were available for 81 patients

^d^≥ 10 mg of prednisone (or equivalent) per day for more than 30 days

^e^Transfer from another ward included transfers from another ICU and from the medical wards

^f^Data were available for 101 patients

^g^Data were available for 79 patients

^h^Mortality was defined as death from any cause within 28 days following the ICU admission

^i^Complicated course was defined as death from any cause within 28 days following the ICU admission and/or mechanical ventilation > 7 days

At ICU admission, median CD4 lymphocyte count was 170 cells/µL [20–430], with 66 patients (54%) equal or below 200 cells/µL (Low-CD4 group) and 57 (46%) above 200 cells/µL (High-CD4 group). Both these groups did not differ regarding demographics, performance status, factors of immunosuppression other than HIV and comorbidity, except for COPD which was more prevalent in the High-CD4 group (*n* = 12 (21%) vs*. n* = 4 (6%), *p* = 0.01).

### Microbiological investigations

The microbiological investigations are displayed in Additional file 1: Table S2. mPCR was performed in NP swabs exclusively (*n* = 46, 37%) or in LRT specimen exclusively (*n* = 50, 41%), or both (*n* = 27, 22%). Respiratory tract specimens for bacterial culture have been obtained in 110 (91%) patients. BAL fluid has been obtained in 77 (63%) patients.

### Causative diagnosis of ARF

Causative diagnoses of ARF are displayed in Table 2. An opportunistic lung infection was diagnosed in 38 (31%) patients. Seven of the 11 patients with newly diagnosed HIV infection and 8 patients receiving ART, but with a poor adherence to the treatment had PCP.

**Table 2:** Causative diagnosis of acute respiratory failure in 123 HIV-infected patients admitted to the ICU

Patients	All patients (*n* = 123)	CD4 ≤ 200 (*n* = 66)	CD4 > 200 (*n* = 57)
*Pneumocystis jirovecii* pneumonia	29 (23.6)	26 (39.4)	3 (5.3)
Other opportunistic lung infection^a^	9 (7.3)	7 (10.6)	2 (3.5)
Non-opportunistic acute lung infection	59 (48)	22 (33.3)	37 (64.9)
Bacteria	53	21	32
*Streptococcus pneumoniae*	14	9	5
*Other Streptococcus*	3	1	2
*Staphylococcus aureus*	9	5	4
*Legionella pneumophila*	3	0	3
*Haemophilus influenzae*	2	0	2
*Moraxella catarrhalis*	1	0	1
*Klebsiella pneumoniae/Escherichia coli*	2	0	2
Other enterobacteria	5	1	4
*Pseudomonas aeruginosa*	9	4	5
Other Gram-negative bacteria	1	1	0
*Mycoplasma pneumoniae*	1	0	1
Anaerobes	1	0	1
Other bacteria	2	0	2
Virus	25	13	12
Rhinovirus	8	6	2
Adenovirus	2	1	1
Coronavirus	1	1	0
Influenza virus	6	2	4
Human metapneumovirus	1	0	1
Parainfluenza virus	3	2	1
Respiratory syncytial virus	4	1	3
Bacteria–virus coinfection	12	8	4
Virus–virus coinfection	2	2	0
Undocumented	8	2	6
Non-infectious lung disease^b^	19 (15.4)	5 (7.6)	14 (24.6)
Extra-pulmonary cause^c^	7 (5.7)	6 (9.1)	1 (1.8)

Data are presented as number (%). CD4 refers to CD4 lymphocyte count (cells/µL)

^a^Other opportunistic lung infections included CMV-associated pneumonia (*n* = 5) and pulmonary tuberculosis (*n* = 4)

^b^Non-infectious lung diseases included acute exacerbation of COPD of non-infectious etiology (*n* = 6), cardiogenic lung edema without underlying lung agent (*n* = 5), cryptogenic hemoptysis (*n* = 1), intra-alveolar hemorrhage (*n* = 1); acute interstitial pneumonia (*n* = 2), Mendelson syndrome (*n* = 1), sickle cell disease (acute chest syndrome) (*n* = 1); neoplastic pleural effusion (*n* = 1) and Castleman disease (*n* = 1)

^c^Extra-pulmonary causes included histoplasmosis (*n* = 1), *Cryptococcus neoformans* meningitis (*n* = 1), bacterial meningitis (*n* = 2), pyelonephritis (*n* = 2) and bacteremia of unknown origin (*n* = 1)

Non-opportunistic acute lung infections were identified as causative diagnosis of ARF in 59 (48%) patients. All the bacteria-infected patients received an appropriate antimicrobial regimen within the first 24 h of ICU stay. Eight patients had a clinical presentation suggestive of lung infection, but without microbiological documentation.

The ARF was attributed to a non-infectious lung disease in 19 (15%) patients, mainly related to a decompensated chronic condition, i.e., acute exacerbation of COPD and pulmonary edema.

### Analysis according to the viral diagnosis

Overall, 36 respiratory viruses were identified in 33 (27%) patients (Table 3). Rhinovirus was predominant (*n* = 15), followed by Influenza (*n* = 6), Respiratory Syncytial Virus (*n* = 6) and Parainfluenza Virus (*n* = 5). Only one pure virus–virus coinfection was found.

**Table 3:** Description of respiratory virus-associated infections and coinfections with non-viral pathogens

Respiratory viruses	Rhinovirus (*n* = 15)	Influenza virus (*n* = 6)	Parainfluenza virus (*n* = 5)	RSV (*n* = 6)	Coronavirus (*n* = 1)	Adenovirus (*n* = 2)	hMPV (*n* = 1)
Coinfection with at least one non-viral pathogen^a^	11	3	3	4	0	2	0
*Streptococcus pneumoniae*	1	2	1	0	0	0	0
*Streptococcus* sp.	0	0	0	1	0	0	0
*Staphylococcus aureus*	2	2	0	0	0	0	0
Enterobacteria	1	0	0	1	0	1	0
*Pseudomonas aeruginosa*	3	0	0	1	0	0	0
*Corynebacterium* sp.	0	0	0	0	0	1	0
*Mycobacterium tuberculosis*	2	0	0	0	0	0	0
*Pneumocystis jirovecii*	4	0	2	2	0	0	0

*RSV* Respiratory Syncytial Virus, *hMPV* human Metapneumovirus

^a^Some viruses had several non-viral copathogens

**Fig. 1:**
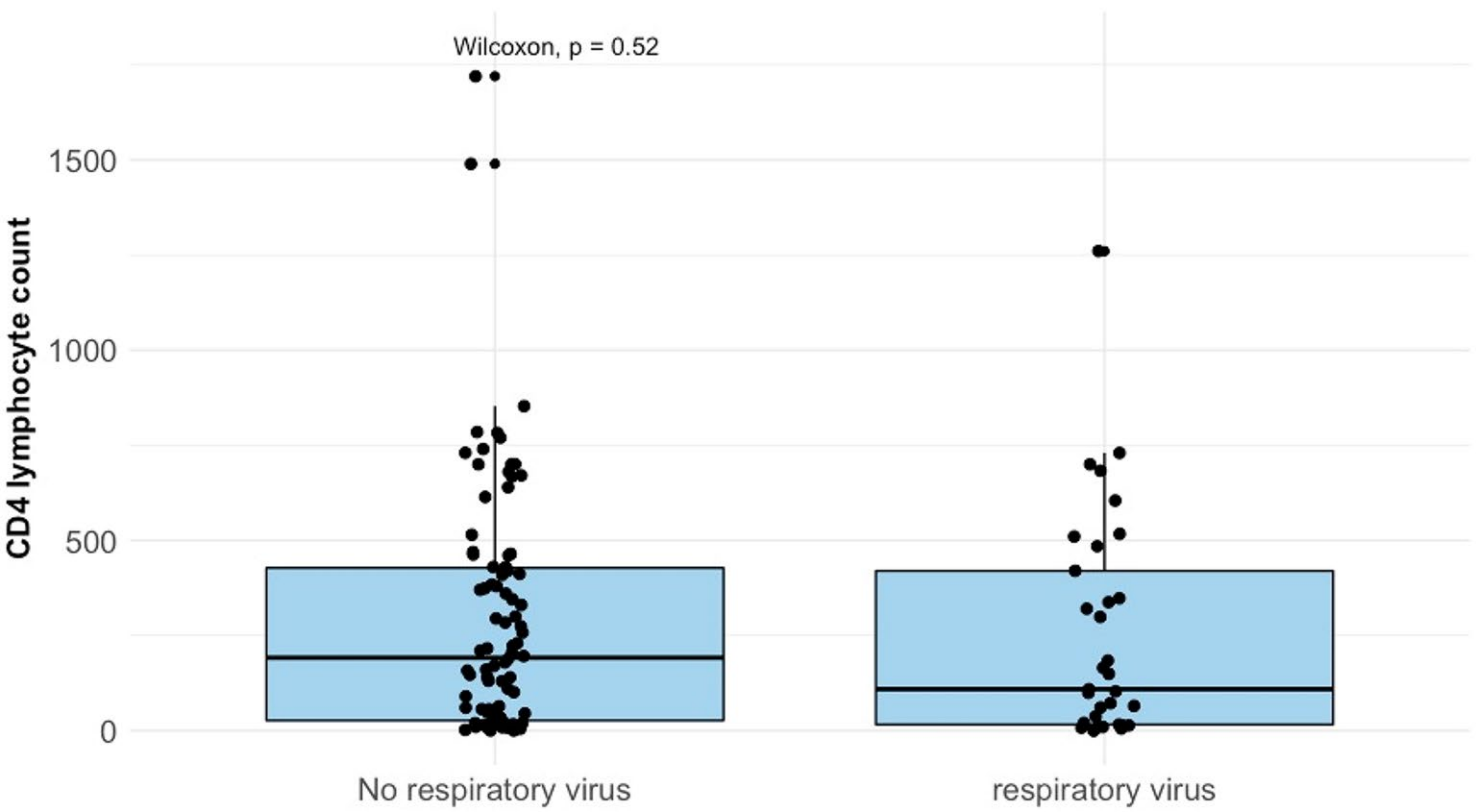
Diagnosis of respiratory virus-associated infection according to the CD4 lymphocyte count on the ICU admission

**Fig. 2:**
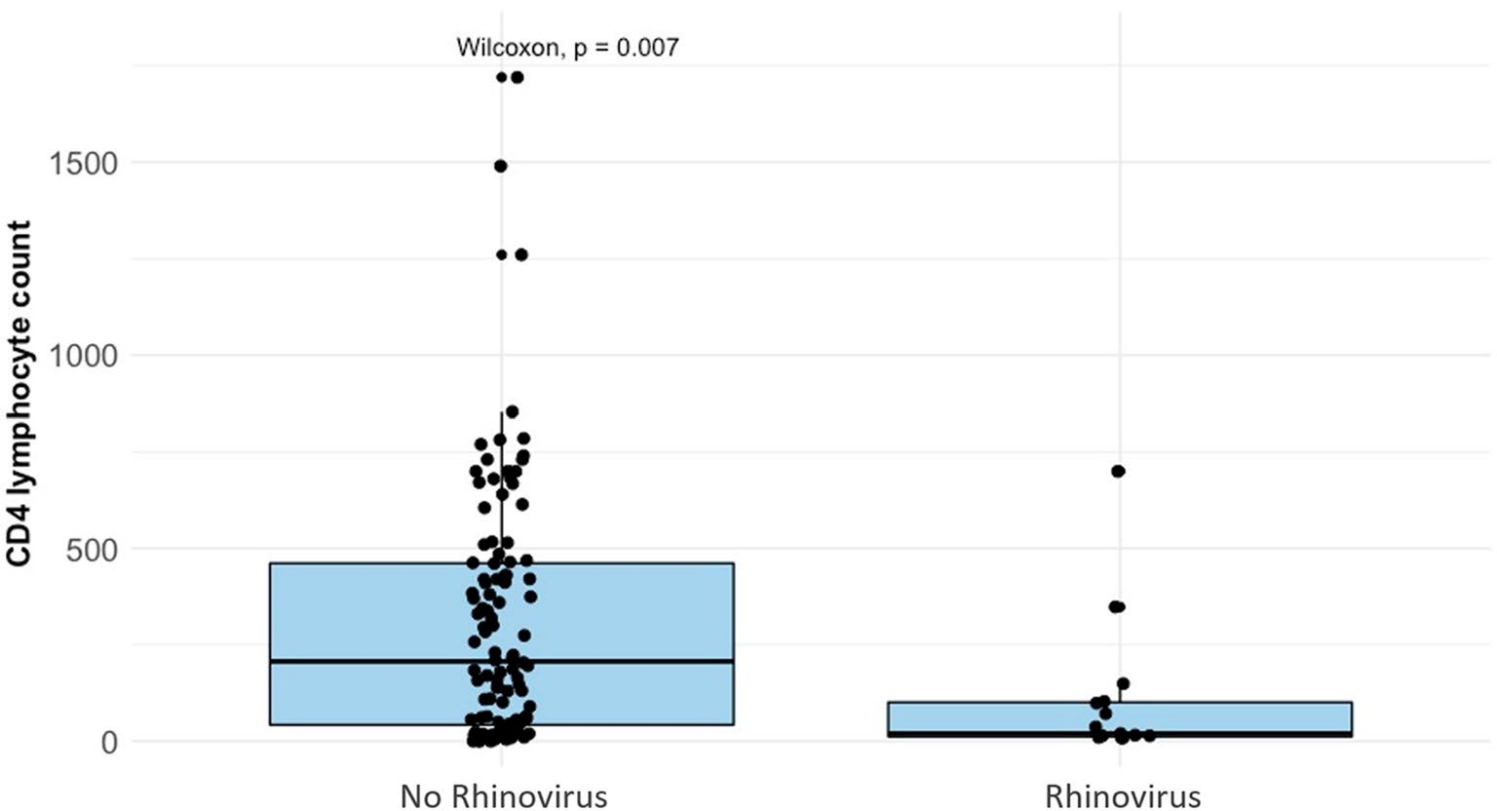
Diagnosis of Rhinovirus-associated infection according to the CD4 lymphocyte count on the ICU admission

The prevalence of respiratory virus-associated infection did not differ among Low- and High-CD4 groups (Table 1); therefore, the median CD4 lymphocyte count in respiratory virus-infected patients was 109 [16–420] cells/µL, in comparison with 192 [27–428] cells/µL in non-infected patients (Fig. 1). However, the prevalence of Rhinovirus-associated infection was higher in the Low-CD4 group, and three-quarters of Rhinovirus-infected patients exhibited a CD4 lymphocyte count below 200 cells/µL (Fig. 2).

In 22 patients, the viral documentation was accompanied by a non-viral documentation (Additional file 1: Figure S1), with bacteria–virus coinfection in 11 patients, bacteria–virus–virus in 2 patients, *P. jirovecii*–virus in 7 patients and *P. jirovecii*–virus–virus in one patient.

The rate of viral documentation among patients explored with NP swab exclusively, LRT specimen exclusively, or both, did not differ significantly (30%, 26% and 22%, respectively; *p* = 0.73).

Documentation of respiratory viruses was more frequent during the winter period (October to March) (Additional file 1: Figure S2). Conversely, Rhinovirus documentation did not follow a seasonal distribution, since only 7/15 were observed during the period from October to March.

Characteristics of the population, stratified by respiratory virus-associated infection are presented in Additional file 1: Table S3. At ICU admission, respiratory virus-infected patients displayed higher respiratory rate and fever. In multivariate analysis, female gender, smoking and steroid therapy were shown as independently associated with respiratory virus-associated infection (Table 4).

**Table 4:** Multivariate analysis of the risk factors for respiratory virus-associated infection in 123 HIV-infected patients admitted to the ICU for acute respiratory failure

Variables	Univariate analysis			Multivariate analysis		
	Odds ratio	95% confidence interval	*p* value	Odds ratio	95% confidence interval	*p* value
Female gender	2.45	[1.07–5.59]	0.03	2.8	[1.1–7.1]	0.03
Smoking	2.1	[0.9–4.8]	0.07	3.6	[1.4–9]	0.007
Steroid therapy	8.9	[0.9–88.8]	0.06	18.3	[1.4–236]	0.03
Maximal temperature^a^	1.4	[1.01–2]	0.04			
Maximal respiratory rate^a^	1.06	[1–1.12]	0.04			
Leucocyte count^a^	0.9	[0.81–0.97]	< 0.01	0.9	[0.7–0.9]	0.004
Neutrophil count^a^	0.9	[0.79––0.98]	0.02			
Lymphocyte count^a^	0.59	[0.33––1.06]	0.08			

^a^Refers to values on the ICU admission

### Outcome

Mortality at Day-28 was 12%, and 26% of patients displayed a complicated course, without difference between High-CD4 and Low-CD4 groups (Table 1). We investigated whether a respiratory virus-associated infection affected prognosis. In the analysis stratified by respiratory virus-associated infection, outcome was similar between infected and non-infected patients (Additional file 1: Table S3). In multivariate analysis, a respiratory virus-associated infection was not identified as an independent factor of either a complicated course (Table 5) or death at Day-28 (Additional file 1: Table S4).

**Table 5:** Multivariate analysis of the risk factors for complicated course in 123 HIV-infected patients admitted to the ICU for acute respiratory failure

	Univariate analysis			Multivariate analysis		
Variables	Odds ratio	95% confidence interval	*p* value	Odds ratio	95% confidence interval	*p* value
Chronic dialysis	3.1	[0.73–13.15]	0.12			
Cirrhosis (Child B–C)	2.76	[0.49–15.65]	0.25			
Cancer or hematologic malignancy	2.76	[0.49–15.65]	0.25			
Use of vasopressor^a^	7.76	[1.71–35.29]	< 0.01	7.2	[1.5–35.4]	0.01
Pleural effusion	2.34	[0.68–8.1]	0.18	3.6	[1.06–12.2]	0.04
HIV viral load^b^	1.14	[0.89–1.45]	0.31			
PaO2/FIO2 ratio^b^	0.995	[0.99–1]	0.05			
Urea^b^	1.035	[1–1.07]	0.06			
Alkaline phosphatase^b^	1.008	[1–1.01]	< 0.01	1.008	[1.002–1.01]	0.006
Minimal platelet count^b^	0.996	[0.99–1]	0.05			
Fibrinogen^b^	0.79	[0.59–1.06]	0.12			
Minimal prothrombin time^b^	0.979	[0.95–1.01]	0.12			

Complicated course was defined as death from any cause within 28 days following the ICU admission and/or mechanical ventilation > 7 days

^a^During the first hour of the ICU stay

^b^Refers to values at ICU admission

## Discussion

This retrospective study investigated the epidemiology of respiratory viruses in HIV-infected adults admitted to the ICU for ARF. Real-time mPCR tests identified at least one virus in the respiratory tract of 27% of patients, but with a non-viral copathogen in two-thirds of cases. The prevalence of respiratory virus-associated infection did not vary along with the level of the CD4 T-cell deficiency, except for Rhinovirus which was more prevalent in patients with a CD4 lymphocyte count below 200 cells/µL. In multivariate analysis, respiratory virus-associated infection was not associated with a worse prognosis.

In this study, more than one patient out of four (27%) were infected with at least one respiratory virus. This finding illustrated the high yield of an aggressive diagnostic strategy with a broad panel mPCR on respiratory tract specimens. Our results are original since prior works having described the etiological panel of ARF in HIV-infected ICU patients were conducted before the era of real-time mPCR [2, 4, 22]. Interestingly, the rate of viral documentation that we observed was similar to what had been described (27 to 29%) previously in non-HIV adults admitted to the ICU for an acute respiratory disorder requiring intubation [7, 8].

We identified at least one non-viral copathogen in more than two-thirds of the patients with a viral documentation, in line with a recent report in a population with a high prevalence of tuberculosis [19, 23]. Non-opportunistic acute lung infections, including pneumonia, acute bronchitis and exacerbation of COPD, were the first cause of ARF, consistent with previous reports in Western countries [2, 4]. This finding highlights the burden of chronic respiratory conditions in aging HIV-infected populations [6]. Here, more than 40% of patients were smokers. Synergistic effects of tobacco and HIV [24] in promoting chronic bronchitis and pro-COPD changes in the lung [25] have been demonstrated. Moreover, high rates of viral documentation within airways of COPD patients both at stable state and during exacerbation have been reported [26]. These data may explain the high rate of viral documentation that we observed. In multivariate analysis, smoking was independently associated with respiratory virus-associated infection. This finding is in line with previous works demonstrating that tobacco exposure alters immune responses to Rhinovirus [27], Influenza Virus [28] and Respiratory Syncytial Virus [29]. Interestingly, female gender was associated with an increased risk of respiratory virus-associated infection on multivariate analysis. Prior cohort studies in primary care described an increased risk for development of Influenza-like illnesses in women compared to men [30, 31]. However, to our knowledge, no prior study has specifically explored this point in HIV-infected populations admitted for ARF.

In this study, we also aimed to investigate a putative role of the HIV-related CD4 T-cell deficiency in promoting respiratory virus-associated infection. Previous studies explored this point in children, but with conflicting results. Annamalay et al. described similar rates of viral documentation in HIV-infected and non-infected children admitted for lower respiratory tract infections [32], whereas O’Callaghan-Gordo et al. observed that respiratory virus-associated infections were 6 to 16 times more prevalent among HIV-infected children admitted for pneumonia [33]. As we did not include a comparative non-HIV population, we rather examined whether or not the rate of viral documentation varied along with the level of CD4 T-cell deficiency. Finally, we found no association between the CD4 lymphocyte count and the risk of respiratory virus-associated infection, in line with a previous report focusing on Influenza viruses [34].

Rhinovirus was the predominant virus, accounting for more than 40% of viral documentations. This high prevalence was consistent with that of previous reports in ICU patients with ARF [7], community-acquired pneumonia [35] or acute exacerbation of COPD [13]. Surprisingly, Rhinovirus was much more prevalent in low-CD4 patients. This finding is original, since no prior work has specifically explored this point in adults. In HIV-infected children, Rhinovirus has been shown highly prevalent, during both pneumonia and bronchiolitis, but without data regarding a putative association with the level of CD4 T-cell deficiency [32, 36]. Other data in hematology and cancer adults demonstrated high rates of Rhinovirus documentation within airways during respiratory tract infections [37, 38]. To explain this high prevalence in immunocompromised patients, a mechanism of prolonged viral shedding has been proposed, rather than iterative reinfections as observed in COPD patients [39]. The prolonged Rhinovirus shedding may be attributable to an inefficient immunological control of a single infectious episode [40, 41]. Therefore, in pediatric hematopoietic stem cell transplant recipients with a persistent Rhinovirus shedding (≥ 30 days), Piralla et al. demonstrated significant lower CD4, CD8 and NK lymphocyte counts at the onset of infection, as compared to children with a brief Rhinovirus shedding. Moreover, a decrease in Rhinovirus load was associated with significant increases of the same lymphocyte counts [42]. These data would suggest an important role for the T-cell immunity in the control of Rhinovirus infection, and subsequently, may explain a delayed Rhinovirus clearance in low-CD4 HIV-infected patients, resulting in persistent shedding and increased prevalence.

We observed a high rate of dual infection, either virus–bacteria or virus–opportunistic pathogen. These findings made us consider the prognostic impact of such coinfections. Studies in ICU adult patients with pneumonia suggested that virus–bacteria coinfection was associated with a worse prognosis [43]. In mice, the coinfection of Influenza with *Streptococcus pneumoniae* [44], *Legionella pneumophila* [45] or *Staphylococcus aureus* [46] impaired the anti-Influenza immune response and increased mortality. Whether similar synergistic effects exist in virus–opportunistic pathogen coinfection remain unknown. Only one animal study has explored the couple *Pneumocystis jirovecii*–Influenza, but in a successive rather than concomitant model [47]. Unfortunately, in our study, the low number of observations prevented us from analyzing the prognosis according to the presence of coinfections.

Our study has several limitations. First, this study included adult patients with ARF that required ICU admission, preventing any conclusion on other populations such as HIV-infected children or HIV-infected adults with ARF that did not require ICU admission. Second, the study was retrospective, so we did not control the microbiological investigations. The preferred sample for mPCR test in non-intubated patients was not the sputum, but the nasopharyngeal swab [48]. Several factors may have discouraged clinicians to use sputum, including the high number of patients unable to produce sputum [49] and the highly viscous nature of this sample that can make nucleic acid extraction difficult [50]. By definition, an mPCR was performed in the respiratory tract of every included patient because it was a criterion for patient screening. But some other microbiological tests were only occasionally performed, i.e., cytomegalovirus PCR. Furthermore, the retrospective design prevented us from obtaining a number of data, which were rarely reported in medical records by physicians, including vaccine history, *Pneumocystis jirovecii* prophylaxis, symptoms before hospital referral, and duration of symptoms before ICU admission. Third, only patients having undergone an mPCR in the respiratory tract within the 72 h following their ICU admission were screened; this might suggest a confounding of indication. Fourth, the choice to classify patients according to their CD4 lymphocyte count on the ICU admission, instead of the latest known value, might be criticized. However, this choice was guided by the high number of missing values in the latest CD4 lymphocyte count as well as the number of newly diagnosed patients without any prior CD4 lymphocyte count. Fifth, we assumed that a virus identified with PCR in nasopharyngeal or lower respiratory tract samples was always a pathogen of the respiratory tract, whatever the clinical picture and radiological features. This might be criticized since respiratory viruses might be present in asymptomatic adult subjects [15], even if it seems rare, about 2% of asymptomatic adults seen at the emergency department [16]. Sixth, to avoid overinterpreting the data, we decided to consider respiratory viruses as a homogeneous group of pathogens in the analysis stratified by respiratory virus-associated infection. This might be criticized since the pathogenicity differs from one viral species to another.

## Conclusions

Viruses are frequently identified in the respiratory tract of HIV-infected patients with ARF that required ICU admission, but with a non-viral copathogen in two-thirds of cases. Rhinovirus is the predominant viral specie; its prevalence is highest in patients with a CD4 lymphocyte count below 200 cells/µL.

## Supplementary information

**Additional file 1.** Additional information on Material and methods, Table S1 (Panels of mPCR kits used in the two participating ICUs over the 6-year study period), Table S2 (Microbiological investigations performed in 123 HIV-infected patients admitted to the ICU for acute respiratory failure, according to the diagnosis of respiratory virus-associated infection), Table S3 (Baseline characteristics, behavior during ICU stay, and outcome of 123 HIV-infected patients admitted to the ICU for acute respiratory failure, according to the diagnosis of respiratory virus-associated infection), Table S4 (Multivariate analysis of the risk factors for death at Day-28 in 123 HIV-infected patients admitted to the ICU for acute respiratory failure), Figure S1 (Distribution of the microbiological documentations in 123 HIV-infected patients admitted to the ICU for acute respiratory failure), Figure S2 (Seasonal distribution of viral documentations).

## Data Availability

Data and materials supporting the findings of this study can be entirely shared if asked.

## Abbreviations

ARF: Acute respiratory failure
BAL: Bronchoalveolar lavage
CMV: Cytomegalovirus
COPD: Chronic obstructive pulmonary disease
ESM: Electronic supplementary material
HIV: Human immunodeficiency virus
ICU: Intensive care unit
LRT: Low respiratory tract
mPCR: Multiple polymerase chain reaction
NP: NasoPharyngeal
PCP: *Pneumocystis jirovecii* pneumonia
SAPS II: Simplified Acute Physiology Score
SOFA: Sepsis-related Organ Failure Assessment
WHO: World Health Organization
