# Supplementary material for: Respiratory virus-associated infections in HIV-infected adults admitted to the intensive care unit for acute respiratory failure: a 6-year bicenter retrospective study (HIV-VIR study)
Source: Ann Intensive Care. 2020 Sep 14;10:123. doi: 10.1186/s13613-020-00738-9 (PMC7488215; doi:10.1186/s13613-020-00738-9)
Supplement: Supplementary file 1 — Additional file 1. Additional information on Material and methods, Table S1 (Panels of mPCR kits used in the two participating ICUs over the 6-year study period), Table S2 (Microbiological investigations performed in 123 HIV-infected patients admitted to the ICU for acute respiratory failure, according to the diagnosis of respiratory virus-associated infection), Table S3 (Baseline characteristics, behavior during ICU stay, and outcome of 123 HIV-infected patients admitted to the ICU for acute respiratory failure, according to the diagnosis of respiratory virus-associated infection), Table S4 (Multivariate analysis of the risk factors for death at Day-28 in 123 HIV-infected patients admitted to the ICU for acute respiratory failure), Figure S1 (Distribution of the microbiological documentations in 123 HIV-infected patients admitted to the ICU for acute respiratory failure), Figure S2 (Seasonal distribution of viral documentations). [file 13613_2020_738_MOESM1_ESM.docx]

**ADDITIONAL MATERIAL**

**Material and Methods**

**Data collection**

Data regarding demographics, comorbidity, World Health Organization Performance status, initial clinical, laboratory and radiological findings (Chest X-ray during the first 24 hours of the ICU stay), microbiological investigations during the ICU stay, therapeutic management during the ICU stay, ICU and hospital lengths of stay, and ICU and hospital discharge status (dead or alive), were collected.

The HIV infection may be either previously known or newly diagnosed (*de novo)* during the ICU stay. HIV-related characteristics were collected, including newly diagnosed HIV infection, time since HIV diagnosis, latest available CD4 lymphocyte count and HIV viral load, ongoing antiretroviral therapy (ART), use of prophylaxis against *Pneumocystis jirovecii,* CD4 lymphocyte count and HIV viral load during the ICU stay.

A transfer from another ward was defined as transfer from another ICU or from the medical wards. We collected the need for mechanical ventilation and its duration, the need for renal replacement therapy and for vasopressors.

Mortality was defined as death from any cause within 28 days following the ICU admission.

**Microbiological evaluation using mPCR**

During the study period, different mPCR kits were used in Bichat Hospital: the Respifinder^TM^ 19 (Pathofinder®, Maastricht, Netherlands) from May 2011 to February 2012, Respifinder^TM^ 22 (Pathofinder®, Maastricht, Netherlands) from March 2012 to December 2014 and Anyplex^TM^ II RV16 (Seegene®, Seoul, South Korea) after January 2015. The FilmArray Respiratory Panel (BioFire Diagnostics®, Salt Lake City, USA) was also used upon specific request. Of note, the Respifinder® 22 and the FilmArray Respiratory panel included four bacterial targets (*L. pneumophila*, *M. pneumoniae*, *C. pneumoniae* and *B. pertussis*) and three bacterial targets (*M. pneumoniae*, *C. pneumoniae* and *B. pertussis*), respectively. During the study period, different mPCR kits were used in Tenon Hospital: Anyplex^TM^ II RV16 (Seegene®, Seoul, South Korea) from 2011 to 2016, Allplex^TM^ Respiratory Panel Assays (Seegene®, Seoul, South Korea) from 2016 to 2017.

**Microbiological evaluation for non-viral pathogens**

Respiratory tract specimens underwent Gram staining and quantitative culture for bacterial pathogen, or Auramine-rhodamine staining and culture for mycobacteria. Urine antigen testing of *Streptococcus pneumoniae* and *Legionella pneumophila* used the BinaxNOW kits (Alere, Jouy en Josas, France). Bronchoalveolar lavage fluid underwent Grocott staining, immunofluorescence and PCR (TaqMan, ThermoFischer Scientific®) for *Pneumoyctis jirovecii*.

Either in blood sample or in bronchoalveolar lavage fluid, the cytomegalovirus (CMV) PCR used the CMV R-gene kit (Argene®) or the QS-RGQ kit (Qiagen®).

**Classification of patients according to the causative diagnosis**

Five mutually-exclusive classes of causative diagnosis for ARF were defined, as followed:

- *Pneumocystis jirovecii* pneumonia (PCP): defined by the documentation of *Pneumocystis jirovecci* in bronchoalveolar lavage (BAL) fluid and/or induced sputum (Grocott staining and/or immunofluorescence and/or PCR);

- Other opportunistic lung infection: defined by the documentation of opportunistic pathogen(s) (*Mycobacterium tuberculosis*, cytomegalovirus (CMV), fungi, others) in lower respiratory tract specimen, but without simultaneous documentation of *Pneumocystis jirovecii*; the diagnosis of CMV-associated pneumonia was retained when both the PCR in blood and BAL fluid were positive.

- Non-opportunistic acute lung infection: defined by the presence of clinical signs of acute lung infection at ICU admission (a recent onset of at least two of the following criteria: body temperature > 37.8°C, chest pain, cough, sputum production, localized crackles with or without pleural syndrome), but without any documentation of opportunistic pathogen; the non-opportunistic acute lung infection may have been either documented (bacteria or virus or bacteria-virus coinfection) or undocumented. A bacteria was considered as causative pathogen of the acute lung infection if this bacteria fulfilled at least one criteria among the followings: i) *S. pneumoniae* or *S. aureus* or *L. pneumophila*, whatever type of testing and level of positivity; ii) identified in pleural fluid or blood; iii) *Chlamydiae pneumoniae* or *Mycoplasma pneumoniae* identified with Ig antibodies testing; iv) *Bordetella pertussis* or *C. pneumoniae* or *M. pneumoniae* identified by mPCR; v) identified within a sputum specimen with good-quality criteria (leucocyte > 25/field and epithelial cells < 10/field) and ≥ 10^6^ colony-forming units/mL); vi) identified within bronchoalveolar lavage fluid at ≥ 10^4^ colony-forming units/mL or protected distal sample at ≥ 10^3^ colony-forming units/mL, or bronchial aspirate specimen at ≥ 10^5^ colony-forming units/mL.

- Non-infectious lung disease: defined by an acute lung disease but without any documentation of opportunistic pathogen and without the clinical signs of acute lung infection; this class included mainly exacerbation of chronic obstructive pulmonary disease (COPD), pulmonary edema, interstitial pneumonia (*de novo* or exacerbation), hemoptysis and diffuse alveolar hemorrhage.

- Extra-pulmonary cause : defined by an ARF that was not associated with any clinical/radiological sign of lung disease.

**Table S1. Panels of mPCR kits used in the two participating ICUs over the 6-year study period.**

|  | Respifinder^TM^ 19 | Respifinder^TM^ 22 | Anyplex^TM^ II RV 16 | Allplex^TM^ Respiratory Panel Assays |
| --- | --- | --- | --- | --- |
| Center | Bichat | Bichat | Bichat-Tenon | Tenon |
| Study period | 2011 - 2012 | 2012 - 2014 | 2011- 2017 | 2016 - 2017 |
| Adenovirus  Coronavirus  229E  OC43  NL63  HKU1  Influenza A  Influenza B  Human Metapneumovirus  Parainfluenza 1, 2, 3, 4  Respiratory Syncytial Virus A  Respiratory Syncytial Virus B  Rhinovirus  Enterovirus  Bocavirus | +  +  +  +  -  +  +  +  +  +  +  +  -  - | +  +  +  +  +  +  +  +  +  +  +  +  +  + | +  +  +  +  -  +  +  +  +  +  +  +  +  + | +  +  +  +  -  +  +  +  +  +  +  +  +  + |

**Table S2. Microbiological investigations performed in 123 HIV-infected patients admitted to the ICU for acute respiratory failure, according to the diagnosis of respiratory virus-associated infection.**

| Patients | All  patients  (n=123) | Virus+  (n=33) | Virus-  (n=90) | P  Value ^a^ |
| --- | --- | --- | --- | --- |
| mPCR in nasopharyngeal swabs | 73 (59.3) | 20 (60.6) | 53 (58.9) | 0.86 |
| mPCR in LRT specimen |  |  |  |  |
| Bronchoalveolar lavage | 67 (54.5) | 17 (51.5) | 50 (55.6) | 0.69 |
| Endotracheal aspirate | 16 (13) | 2 (6.1) | 14 (15.6) | 0.23 |
| Bacterial culture of respiratory sample |  |  |  |  |
| Sputum | 56 (45.5) | 23 (69.7) | 33 (36.7) | 0.001 |
| Distal protected sample | 13 (10.6) | 1 (3 %) | 12 (13.3) | 0.18 |
| Bronchial aspirate | 32 (29) | 10 (31.3) | 22 (28.2) | 0.75 |
| Bronchoalveolar lavage | 76 (61.8) | 20 (60.6) | 56 (62.2) | 0.87 |
| CMV PCR in BAL fluid  CMV PCR in blood sample | 62 (50.8)  62 (50.8) | 17 (51.5)  18 (54.5) | 45 (50.6)  44 (49.4) | 0.93  0.62 |
| *S. pneumoniae* urine antigen testing | 87 (71.3) | 21 (63.6) | 66 (73.3) | 0.25 |
| *L. pneumophila* urine antigen testing | 89 (72.4) | 23 (69.7) | 66 (73.3) | 0.69 |
| Blood culture | 112 (93.3) | 32 (97) | 88 (97.8) | 0.11 |

Patients were classified as *Virus+* if they had at least one documentation of respiratory virus in the respiratory tract, regardless of the type of specimen (nasopharyngeal or lower respiratory tract).

Data are presented as number (%). BAL = Bronchoalveolar Lavage; LRT = Lower Respiratory Tract; (m)PCR = (multiplex) Polymerase Chain Reaction.

^a^ P values refer to differences between *Virus*+ and *Virus-* groups in univariate logistic regression.

**Table S3. Baseline characteristics, behavior during ICU stay, and outcome of 123 HIV-infected patients admitted to the ICU for acute respiratory failure, according to the diagnosis of respiratory virus-associated infection.**

| Patients | All patients (n=123) | Virus+  (n=33) | Virus-  (n=90) | p  value ^a^ |
| --- | --- | --- | --- | --- |
| Age, y | 51 [42.5-58.5] | 47 [40-56] | 52,5 [43-59] | 0.12 |
| Sex male | 82 (66.7) | 17 (51.5) | 65 (72.2) | 0.03 |
| Smoking | 49 (41.2) | 18 (54.5) | 31 (36) | 0.07 |
| WHO Performans Status > 0 | 61 (50.8) | 14 (45.1) | 47 (52.8) | 0.46 |
| COPD GOLD III-IV  Arterial hypertension  Coronary heart disease  Heart failure (NYHA III-IV)  Cirrhosis (Child B-C)  Chronic dialysis | 16 (13)  32 (26)  19 (15.4)  7 (5.7)  6 (4.9)  9 (7.3) | 4 (12.1)  6 (18.2)  5 (15.2)  2 (6.1)  2 (6.1)  4 (12.1) | 12 (13.3)  26 (28.9)  14 (15.6)  5 (5.6)  4 (4,4)  6 (6.7) | 0.86  0.23  0.96  0.91  0.71  0.22 |
| Baseline HIV-related characteristics  Newly diagnosed HIV infection  HIV viral load (log) ^b^  CD4 lymphocyte count (cells/µL) ^c^  ART | 11 (8.9)  0 [0-3.4]  351 [140-600]  88 (72.1) | 1 (3)  1.5 [0-4.8]  329 [72-513]  27 (81.8) | 10 (11.1)  0 [0-2.3]  390 [183-612]  61 (68.5) | 0.16  0.03  0.39  0.15 |
| Steroid therapy  Other immunosuppressive treatment  Splenectomy  Cancer or hematologic malignancy  Chemotherapy  Organ/bone marrow transplantation | 4 (3.3)  2 (1.6)  1 (0.8)  6 (4.9)  2 (1.6)  1 (0.8) | 3 (9.1)  2 (6.1)  1 (3)  1 (3)  0 (0)  1 (3) | 1 (1.1)  0 (0)  0 (0)  5 (5.6)  2 (2.2)  0 (0) | 0.03  0.02  0.10  0.56  0.39  0.10 |
| Transfer from another ward ^d^ | 62 (50.4) | 21 (63.6) | 41 (45.6) | 0.08 |
| SOFA score  SAPS II score | 4 [2-7]  44 [34.3-56.8] | 5 [2-7.8]  44 [32.5-50.5] | 3 [2-6.3]  44 [35-57] | 0.59  0.88 |
| Clinical signs on ICU admission  Maximal body temperature (°C)  Maximal respiratory rate (min^-1^) | 38 [37.2-39]  30 [26-40] | 38.5 [37.7-39.5]  35 [30-41] | 37.8 [37.1-38.5] 30 [25-38] | 0.04  0.02 |
| Biology on ICU admission  HIV viral load (log) ^e^  CD4 lymphocyte count (cells/µL)  Lymphocyte count (G/L)  Neutrophil count (G/L)  Platelet count (G/L)  Fibrinogen (G/L)  Procalcitonin (µg/L) ^f^  Lactate dehydrogenase (U/L) | 2.5 [0-5.3]  170 [20-430]  0.9 [0.4-1.4]  6.7 [3.9-9]  201 [139-284]  5.8 [4.3-7.3]  0.6 [0.1-6.6]  403 [276-643] | 2.8 [0-5.4]  109 [16-420]  0.6 [0.3-1.1]  5.5 [2.4-7.5]  195 [81-236]  5.3 [3.9-7]  0.4 [0.2-2.3]  385 [246-626] | 2.4 [0-5.3]  192 [27-428]  0.9 [0.5-1.5]  7 [4.6-10.1]  205 [144-288]  6 [4.8-7.4]  0.6 [0.1-8.3]  405 [278-659] | 0.76  0.52  0.02  0.02  0.20  0.18  0.60  0.61 |
| Organ supports during ICU stay  High flow nasal cannula oxygen  Non invasive ventilation  Mechanical ventilation  Vasopressor  Renal replacement therapy | 36 (29.2)  30 (24.8)  43 (35.2)  36 (29.3)  23 (18.7) | 12 (36.3)  7 (21.2)  11 (33.3)  10 (30.3)  7 (21.2) | 24 (26.7)  23 (26.1)  32 (36)  26 (28.9)  16 (17.8) | 0.30  0.58  0.79  0.88  0.67 |
| Outcome  ICU length of stay, day  Day-28 Mortality ^g^  Complicated course ^h^ | 7 [4-12]  11 (8.9)  30 (24.4) | 6 [5-12]  2 (6.1)  7 (21.2) | 8 [4-13]  9 (10)  23 (25.6) | 0.94  0.50  0.62 |

Patients were classified as *Virus+* if they had at least one documentation of respiratory virus in the respiratory tract, regardless of the type of specimen (nasopharyngeal or lower respiratory tract).

Data are presented as median [first through third quartiles] or number (%). SAPS II= Simplified Acute Physiologic Score II; SOFA = Sepsis-related Organ Failure Assessment.

^a^ P values refer to differences between *Virus+* and *Virus-* groups in univariate logistic regression. ^b^ Data were available for 76 patients. ^c^ Data were available for 81 patients. ^d^ Transfer from another ward included transfers from another ICU and from the medical wards. ^e^ Data were available for 101 patients. ^f^ Data were available for 79 patients.  ^g^ Mortality was defined as death from any cause within 28 days following the ICU admission. ^h^ Complicated course was defined as death from any cause within 28 days following the ICU admission and/or mechanical ventilation > 7 days.

Table S4. Multivariate analysis of the risk factors for death at Day-28 in 123 HIV-infected patients admitted to the ICU for acute respiratory failure.

|  | Univariate analysis | | | Multivariate analysis | | |
| --- | --- | --- | --- | --- | --- | --- |
| Variables | Odds  ratio | 95%  confidence  interval | p value | Odds  ratio | 95%  Confidence  interval | p value |
| Chronic dialysis | 7.49 | [1.75-32.07] | <0.01 | 9.8 | [2.1-44.4] | 0.003 |
| Cirrhosis (Child B-C) | 8.7 | [1.59-48.29] | 0.01 |  |  |  |
| Use of vasopressor ^a^ | 4.21 | [0.93-19.04] | 0.06 |  |  |  |
| HIV Viral load ^b^ | 1.12 | [0.98-1.29] | 0.10 |  |  |  |
| Alkaline phosphatase ^b^ | 1.007 | [1-1.01] | <0.01 | 1.007 | [1.002-1.01] | 0.006 |
| Minimal platelet count ^b^ | 0.995 | [0.99-1] | 0.08 |  |  |  |

Death at Day-28 referred to death from any cause within 28 days following the ICU admission

^a^ During the first hour of the ICU stay. ^b^ Refers to values at ICU admission.

Figure S1. Distribution of the microbiological documentations in 123 HIV-infected patients admitted to the ICU for acute respiratory failure.


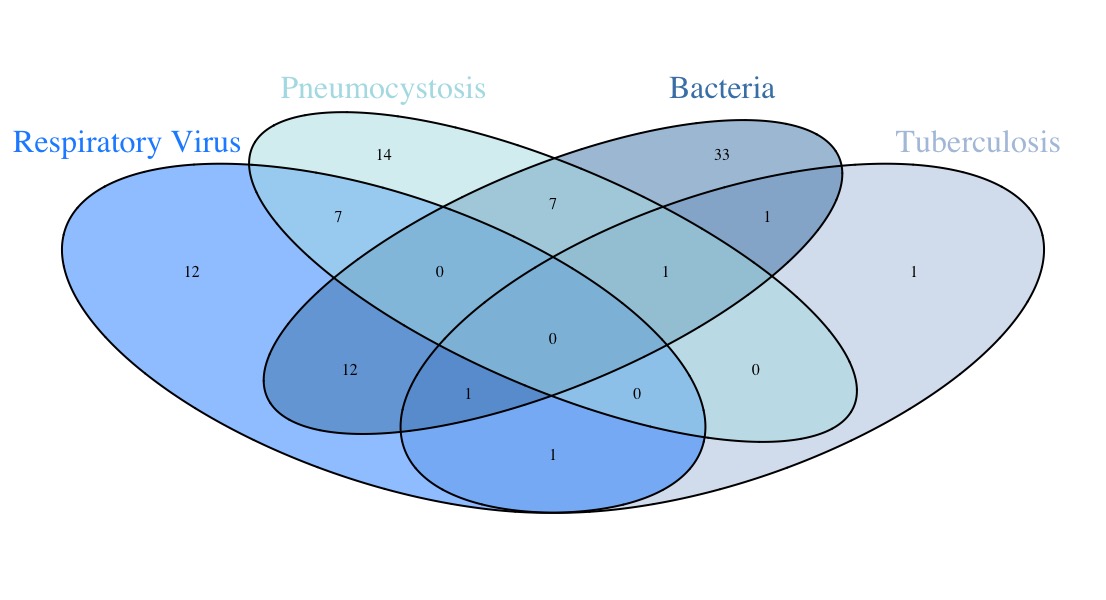


Figure S2. Seasonal distribution of viral documentations.


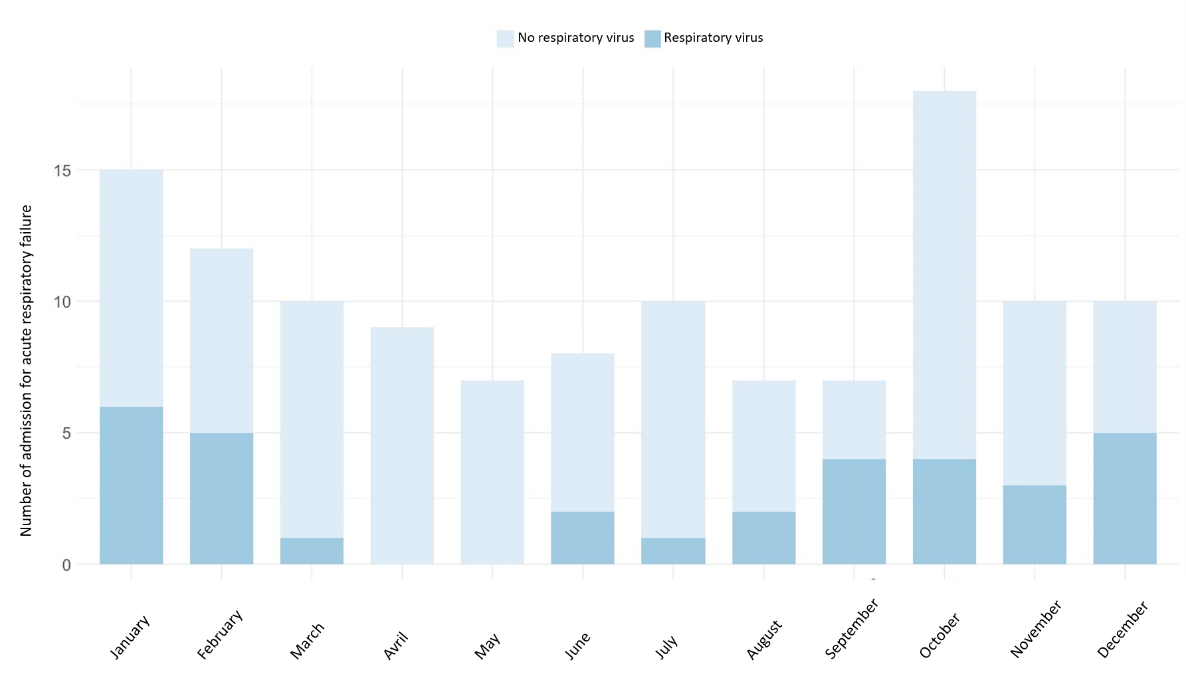


For each month, one bar represents the sum of the cases over the 6-year period of the study.
